# Biomarkers for immunotherapy resistance in non-small cell lung cancer

**DOI:** 10.3389/fonc.2024.1489977

**Published:** 2024-12-19

**Authors:** Catriona Rother, Tom John, Annie Wong

**Affiliations:** ^1^ Wellington Blood and Cancer Centre, Te Whatu Ora Capital, Wellington, New Zealand; ^2^ Department of Medical Oncology, Peter MacCallum, Cancer Centre, Melbourne, VIC, Australia; ^3^ Department of Medicine, University of Otago, Wellington, New Zealand

**Keywords:** non-small cell lung cancer (NSCLC), immunotherapy resistance, precision oncology strategies, biomarkers, circulating biomarkers

## Abstract

Immunotherapy has revolutionised the treatment landscape of non-small cell lung cancer (NSCLC), significantly improving survival outcomes and offering renewed hope to patients with advanced disease. However, the majority of patients experience limited long-term benefits from immune checkpoint inhibition (ICI) due to the development of primary or acquired immunotherapy resistance. Accurate predictive biomarkers for immunotherapy resistance are essential for individualising treatment strategies, improving survival outcomes, and minimising potential treatment-related harm. This review discusses the mechanisms underlying resistance to immunotherapy, addressing both cancer cell-intrinsic and cancer cell-extrinsic resistance processes. We summarise the current utility and limitations of two clinically established biomarkers: programmed death ligand 1 (PD-L1) expression and tumour mutational burden (TMB). Following this, we present a comprehensive review of emerging immunotherapy biomarkers in NSCLC, including tumour neoantigens, epigenetic signatures, markers of the tumour microenvironment (TME), genomic alterations, host–microbiome composition, and circulating biomarkers. The potential clinical applications of these biomarkers, along with novel approaches to their biomarker identification and targeting, are discussed. Additionally, we explore current strategies to overcome immunotherapy resistance and propose incorporating predictive biomarkers into an adaptive clinical trial design, where specific immune signatures guide subsequent treatment selection.

## Introduction

Immunotherapy has revolutionised the treatment landscape of non-small cell lung cancer (NSCLC), offering new hope to patients with advanced disease. Despite impressive improvements in survival outcomes, however, most patients obtain limited long-term benefits from immune checkpoint blockade. Whilst some do not respond at all (primary resistance), others experience disease progression after an initial period of benefit (acquired resistance). Additionally, immunotherapy exposes patients to the risk of potentially serious immune-related adverse events.

Biomarkers that accurately predict immunotherapy resistance are essential for individualising treatment strategies, improving survival outcomes, and minimising potential harms. These purported biomarkers may provide valuable insights into the mechanisms driving immunotherapy resistance—and may include genetic, epigenetic, proteomic, and metabolomic signatures that reveal the complex interplay between the tumour microenvironment and immune system.

Novel biomarkers that further characterise the composition of the tumour microenvironment, as well as the tumour immune profile and microbiome, hold promise for extending current predictive biomarkers beyond programmed death ligand 1 (PD-L1) expression and tumour mutational burden (TMB). Advancing our understanding of these biomarkers not only improves the prediction of resistance but also identifies potential targets for novel therapeutic interventions to overcome resistance and enhance the efficacy of existing immunotherapy agents.

This review article provides a comprehensive overview of the current status of biomarkers for immunotherapy resistance in NSCLC. It explores the underlying mechanisms of resistance and discusses the most important biomarkers in the field. Additionally, it highlights the potential clinical applications of these biomarkers and considers novel approaches to their identification and targeting.

## Mechanisms of immunotherapy resistance

Phase III randomised studies in non-oncogene-driven NSCLC typically report durable responses in 20%–30% of patients treated with immune checkpoint inhibitors (ICI) targeting PD-1/PD-L1 (1). In order to identify clinically useful predictive biomarkers, it is essential to first consider the complex and interdependent mechanisms underlying immunotherapy resistance.

In addition to the temporal classification of immunotherapy resistance (primary/innate vs. secondary/acquired), a third category of “adaptive immune resistance” has also been described, in which cancer is recognised by the immune system but evades attack by employing an escape mechanism. Clinically, this can manifest as primary or secondary resistance, or a mixed response (2). Immunotherapy resistance can also be categorised spatially, classifying mechanisms of immune evasion as those that occur within a tumour cell itself (tumour-cell intrinsic) and those occurring elsewhere (tumour-cell extrinsic) (3).

A 2023 review article by Zhou and Yang provides a concise summary of the incidence of immunotherapy resistance in NSCLC studies (4). Primary immunotherapy resistance has been estimated to lie between 21% and 27% for NSCLC treated with either single-agent or combination ICI in the first-line setting (5–7), and 41% and 44% in second-line management (8, 9). A lower incidence of primary resistance (between 7% and 11%) has been observed when ICI and chemotherapy are administered as a first-line combination treatment strategy (10–12). Secondary immunotherapy resistance affects a greater proportion of NSCLC patients, with an incidence estimated between 52% and 57% in the first-line setting and 32% and 64% in second-line management (13).

Chen and Mellman describe an effective cancer-immunity cycle (CIC) as comprising a number of critical steps for immunotherapy to be effective: release and recognition of tumour cell antigens, T-cell priming and activation, T-cell migration and infiltration into tumours and their surrounding microenvironment, and immune-mediated tumour cell killing (14). Alterations in any of these key processes can facilitate tumour immune evasion and immunotherapy resistance (15) (**Table 1**).

**Table 1:** Mechanisms of immunotherapy resistance.

Cancer cell-intrinsic resistance		Cancer cell-extrinsic resistance	
Mechanism	Example	Mechanism	Example
Tumour neoantigens	• Loss or downregulation	Tumour microenvironment (TME)	• Increased immunosuppressive cells (e.g., Tregs, MDSCs, TAMs) • Increased immunosuppressive molecules/cytokines (e.g., IL-6, IL-8, CXCL10) • Reduced effector T cells (TILs, e.g., CD8+ and CD4+ T cells, B cells, NK cells, dendritic cells) • Altered metabolic landscape (e.g., reduced availability of substrate/growth factors, aerobic glycolysis, acidic environment)
Deficiencies in antigen presentation and recognition	• B2M deficiency • Loss of MHC heterozygosity		
Aberrant intracellular signalling	• Reduced IFN/JAK/STAT signalling • Increased Wnt/*β*-catenin signalling		
Altered expression of immune checkpoints	• Low or absent PD-L1 expression • Increased TIM-3 or LAG-3 expression		
Driver gene mutations	• *EGFR* mutation • *ALK* rearrangements	Effector T Cells	• Dysfunction or exhaustion

### Cancer cell-intrinsic resistance

Many intrinsic resistance mechanisms often stem from aberrations in the CIC. Alterations in cancer cell signalling, immune checkpoint expression, and specific genetic mutations can also contribute to intrinsic immunotherapy resistance.

Tumour neoantigens are newly formed antigens generated by tumour cells as a result of specific alterations, such as somatic mutations, dysregulated RNA splicing, and post-translational modification. These neoantigens are recognised as non-self, potentially triggering an immune response as they are not subjected to central or peripheral tolerance. The repertoire of tumour neoantigens is crucial in activating the immune response and recruiting effector T cells to the TME. Loss or downregulation of these immunogenic neoantigens represents an important mechanism of both innate and acquired immunotherapy resistance. This can occur as a result of genetic or epigenetic alterations that influence their structure, processing, and presentation (16, 17). A study analysing matched pre-treatment and immunotherapy-resistant NSCLC tumour samples, for example, identified genomic changes in resistant clones, resulting in downregulation of key mutation-associated neoantigens and changes in T-cell receptor clonality (18).

Following the transcription and translation of a mutated gene within a tumour cell, the resulting neoantigens are captured by dendritic cells and presented to T cells via major histocompatibility complexes (MHC). This process initiates T-cell priming and anti-tumour immune responses (19). Beta-2-microglobulin (B2M) plays an essential role in the stability and surface expression of MHC class I molecules, making it a critical component of the neoantigen presentation process (17). Impairments in antigen presentation machinery, such as genetic or epigenetic alterations leading to B2M deficiency or loss of MHC heterozygosity, have been associated with immune evasion and both primary and acquired ICI resistance (20, 21). Furthermore, tumour-derived inhibitory molecules, including IL-6, IL-10, and TGF-β, can negatively impact the growth, maturation, and differentiation of dendritic cells, providing another contribution to potential ICI resistance (15).

Anti-PD-1/PD-L1 ICI efficacy is reliant on PD-1/PD-L1 activity, which can be influenced by various factors. Low or absent PD-L1 expression is a key resistance mechanism contributing to both innate and acquired ICI resistance, and this will be discussed in depth in the next section. Additionally, there is growing research interest in the compensatory upregulation of alternative immune checkpoint receptors following ICI treatment, such as T-cell immunoglobulin mucin 3 (TIM-3) and lymphocyte activation gene protein 3 (LAG-3). Altered expression of these alternative checkpoint receptors provides cancer cells an additional mechanism to evade immune detection and has been associated with both adaptive and acquired immunotherapy resistance in NSCLC (22).

Alterations in tumour cell signalling pathways have also been implicated in NSCLC immunotherapy resistance. These recognised pathways play a crucial role in maintaining immunosuppressive properties within the tumour microenvironment, recruiting and differentiating immune-suppressing cells, and facilitating immune evasion.

Interferon-gamma (IFN-γ)—a cytokine secreted by activated T cells—regulates immune responses via downstream Janus kinase (JAK) enzymes and signal transducer and activators of transcription (STATs). This signalling axis is believed to be of primary importance in mediating both primary and acquired ICI resistance (23). Deficiencies in this pathway—including gene mutations, loss of protein expression, negative regulator presence, and epigenetic silencing—can reduce T-cell infiltration, disrupt antigen presentation, and alter PD-L1 expression, thereby reducing the efficacy of ICI blockade (3, 24). In NSCLC, increased production of IFN in the TME has been shown to induce PD-L1 expression on tumour cells, increase the production of tumour antigens, and facilitate immune escape (25).

Amplified signalling in the PI3K/AKT/mTOR pathway can promote tumour cell survival, proliferation, and metabolic adaptations that undermine anti-tumour immune responses. This typically occurs via increased extrinsic signalling mechanisms or reduced expression of negative regulators such as phosphatase and tensin homolog (*PTEN*) (26). Downregulation and loss-of-function *PTEN* mutations have been shown to result in hyperactivation of PI3K/AKT signalling, increased production of immunosuppressive cytokines (such as IL-6 and IL-10), and increased PD-L1 expression, thus promoting ICI resistance (26, 27). In NSCLC, mutations in *EGFR* and *KRAS* have been shown to result in uncontrolled activation of PI3K/AKT/mTOR signalling, remodelling the TME, driving PD-L1 expression, and diminishing the impact of ICIs (28).

Finally, Wnt/*β*-catenin signalling plays an essential role in stem cell pluripotency, lymphocyte development, and tissue homeostasis (29). In malignancy, persistent activation of these pathways, resulting in increased levels of *β*-catenin, appears to have an inverse correlation with infiltration of TILs in the TME, resulting in a non-T-cell inflamed microenvironment and facilitating ICI resistance (26). In NSCLC, increased Wnt/*β*-catenin signalling has been correlated with higher TMB and lower PD-L1 expression (30, 31).

### Cancer cell-extrinsic resistance

Extrinsic resistance mechanisms encompass reduced T-cell functionality, as well as changes in the tumour microenvironment and the host microbiome.

Any factors that affect the balance of different immune and non-immune cell types within the TME have the potential to influence ICI efficacy. The different cellular compartments within the TME will be further explored in a later section where their contribution to immunotherapy resistance will be considered along with their potential clinical applications. Additionally, the secretion of immunosuppressive molecules and cytokines within the TME can further attenuate anti-tumour immune responses by restricting T-cell infiltration and ICI efficacy (15). The metabolic landscape within the TME also plays a role in immunotherapy resistance. The competitive uptake of glucose, amino acids, and growth factors, along with the tumour cell’s preference for aerobic glycolysis and an acidic environment, not only support tumour cell growth and development but also hinder normal immune cell function (32).

Factors that influence T-cell activation, multiplication, and differentiation, such as adverse TME conditions, can result in reduced T-cell effector function and contribute to extrinsic immunotherapy resistance. T-cell exhaustion can occur when T cells are exposed to persistent neoantigens, resulting in a progressive loss of proliferative and functional capacity, including cytokine production and cytotoxic activity. Exhausted T cells typically express high levels of inhibitory receptors, such as PD-1, CTLA-4, TIM-3, and LAG-3. ICIs target these inhibitory receptors to block the signalling pathways responsible for T-cell exhaustion, with the aim of rejuvenating exhausted T cells and restoring their anti-tumour activity. However, not all exhausted T cells respond to ICIs, and the extent of exhaustion can influence the effectiveness of immunotherapy response (23, 33).

Advancing our understanding of the molecular mechanisms of immunotherapy resistance has led to the identification and clinical assessment of potential resistance biomarkers. Many of these mechanisms will be explored in greater detail throughout this article as we consider targetable strategies to prevent and overcome ICI resistance, ultimately aiming to improve outcomes for NSCLC patients.

## Biomarkers of immunotherapy resistance

The two most clinically established biomarkers in the management of NSCLC are PD-L1 and TMB.

### PD-L1 expression

The interaction between the immune checkpoint receptor, PD-1, and protein-ligand, PD-L1, plays a pivotal role in downregulating the immune response and promoting self-tolerance (26). Tumour cells can exploit this interplay by upregulating PD-L1 expression to suppress anti-cancer immune activity. This immunosuppressive PD-1/PD-L1 axis can be blocked via immune checkpoint inhibition (ICI), with 15 different ICI agents currently holding regulatory approval in the treatment of lung cancer (34, 35).

PD-L1 expression is detected by immunohistochemistry (IHC) and quantified most commonly as a “tumour proportion score” (TPS), based on the intensity and percentage of tumour cells demonstrating membranous PD-L1 staining. As a predictive biomarker, PD-L1 holds particular value in the treatment of NSCLC, where the estimated rates of high PD-L1 expression (defined as TPS > 50%) range between 20% and 30% (34). The ability of PD-L1 to predict immunotherapy response and survival outcomes is well established, with increasing PD-L1 expression typically conferring improved ICI response rates and superior survival. Conversely, low or negative PD-L1 expression (accounting for 30%–40%, and 30%–50% NSCLC, respectively) is more commonly associated with immunotherapy resistance (7, 36–42). At present, PD-L1 is the only biomarker used routinely in clinical practice to guide immunotherapy treatment decisions in NSCLC, with many oncological guidelines now suggesting a chemotherapy-sparing treatment strategy for high PD-L1 expressing disease. Both NCCN and ESMO, for example, recommend anti-PD-1/PD-L1 as first-line monotherapy for metastatic NSCLC with PD-L1 TPS ≥ 50% in the absence of targetable mutations, and in combination with chemotherapy for PD-L1 TPS 1%–49% (43, 44).

PD-L1 testing by IHC is affordable, efficient, and relatively uncomplicated to perform; however, this biomarker has several limitations. Most importantly, using PD-L1 expression alone to screen for immunotherapy benefits is not definitive. Some NSCLC cases with high PD-L1 expression may not respond to immunotherapy, whilst some patients with low or PD-L1-negative disease may still respond (8, 45, 46). For instance, CheckMate-017 (47), CheckMate-057 (48), and OAK (9) reported benefits of immunotherapy regardless of PD-L1 expression, raising questions about the overall utility of PD-L1 as a discriminatory biomarker for excluding patients from treatment. Secondly, various PD-L1 assays have been developed, each with disparate staining protocols and antibodies, leading to variability across studies and laboratories. In an attempt to address the lack of standardisation across different platforms, the IASLC BluePrint IHC Comparability Project identified three assays (22C3, 28-8 and SP263) with comparable TPS scoring (49). Nevertheless, no unified standard for defining PD-L1 positivity currently exists, with different cut-off values being used in various trials (34). Thirdly, PD-L1 expression is both spatially and temporally heterogenous. Expression levels have been shown to vary depending on the location of tissue sample (e.g., primary tumour vs. metastatic site, biopsy tissue vs. surgically resected disease, between one metastatic site and another, or even between different locations within the same tumour deposit), potentially resulting in false-negative reporting of PD-L1 status (34, 50). Furthermore, PD-L1 expression is known to be dynamic over the course of disease and in response to different therapeutics, meaning that a treatment-naïve diagnostic biopsy may not be indicative of PD-L1 expression at the time immunotherapy is deployed (50, 51). Finally, there is evidence suggesting that the predictive role of PD-L1 beyond non-squamous NSCLC may be limited (52–54).

Evaluating PD-L1 expression has become an integral component of personalising immunotherapy in the treatment of NSCLC. Alone, however, PD-L1 is not sufficiently accurate or reliable to offer predictive certainty as a biomarker. A 2023 ASCO publications review article references a retrospective study of 45 FDA-approved immunotherapy clinical trials, which found PD-L1 expression to be predictive in only 29% of cases, lacking predictive value in 53%, and not tested in the remaining trials (23). Given these limitations, it is becoming increasingly important to identify additional biomarkers that can be utilised independently or incorporated into composite predictive models to enhance PD-L1’s predictive accuracy.

### Tumour mutational burden

TMB refers to the number of somatic non-synonymous mutations within a tumour’s genome, typically expressed in mutations per megabase (muts/Mb). A higher mutational burden is thought to result in the greater genesis of tumour neoantigens, allowing heightened recognition of malignant cells as “non-self” and a more effective anti-tumour immune response (19, 50).

Numerous studies have reported a correlation between TMB and ICI response in NSCLC populations. Generally, a high TMB is associated with greater and more durable immunotherapy responses, as well as improved survival outcomes—whereas a low TMB typically confers ICI resistance (5, 55–59). Furthermore, the value of high TMB as a favourable biomarker of immunotherapy response and survival has been noted across PD-L1 expression levels and has been able to independently predict outcomes irrespective of microsatellite instability (MSI) status or underlying tumour type (60, 61). Having said this, it is worth noting that the ability of high TMB to predict long-term clinical benefit is somewhat controversial, with inconsistent OS data reported in CheckMate-227, for example (34).

Different testing platforms can be utilised to assess tumoural TMB, including whole genome sequencing (WGS), whole exome sequencing (WES), and targeted panel sequencing via next-generation sequencing (NGS). TMB is most accurately assessed by WES; however, this modality is costly, time-consuming, and requires comparatively large tissue samples. As such, panel-based NGS sequencing has emerged as a more practical application for clinical use, and numerous different panels have now been developed and verified (19, 62). Thus far, the US FDA has approved three NGS panels for tissue TMB (tTMB) assessment: FoundationOne CDx, TSO 500, and MSK-IMPACT. The utility and application of blood-based TMB (bTMB) will be discussed elsewhere.

Akin to PD-L1, TMB faces a number of limitations as a predictive biomarker. Most importantly, a high TMB status does not universally result in superior immunotherapy outcomes, and similarly, a low TMB status is unable to definitively exclude any ICI response—representing a significant flaw in TMB’s predictive capabilities as an independent biomarker (5, 63–65). Secondly, considerable variation exists across differing TMB testing platforms—with disparate panel sequence sizes, mutation types and numbers, sample requirements, and output capabilities—and no standardisation between different analytical methods (19). Thirdly, other than CheckMate-227, much of the data to support the use of TMB in NSCLC is exploratory or retrospective—as succinctly summarised in a 2022 IASLC review article (34). Finally, there is currently no clear threshold to determine “high TMB” status that is consistently able to predict immunotherapy resistance. Varying thresholds have been utilised across different clinical trials, and the FDA-recommended cut point of ≥ 10 muts/Mb has been challenged in multiple studies (66). In fact, it seems highly unlikely that a specific TMB threshold will ever be able to reliably predict clinical benefit from immunotherapy across differing disease subtypes and stages.

In June 2020, the US FDA granted its first tumour-agnostic approval for second-line pembrolizumab in the treatment of any unresectable or metastatic solid tumour with high tTMB—defined as ≥ 10 muts/Mb, as determined by the FoundationOne CDx assay—where no satisfactory alternative therapies exist (67). This decision followed the results of KEYNOTE-158, a multi-cohort phase II trial, in which high tTMB was associated with improved ORR (29% vs. 6%) across 10 different malignancy types treated with pembrolizumab, regardless of PD-L1 expression (68). It is worth noting that this study contained small-cell lung cancer patients, but no NSCLC cohort. Importantly, a recently published guideline from a collective group including the IASLC and College of American Pathologists recommended that clinicians should not use TMB alone to select patients with advanced NSCLC for ICI therapy, based on insufficient evidence in this population (69).

## Emerging biomarkers

To date, the translation of successful immunotherapy combinations from preclinical models into the clinic has been hindered by the lack of robust biomarkers. Early biomarker studies have separated tumours into “cold” and “hot” tumour microenvironments based on the presence of T cells and PD-L1 expression. Functional studies using RNA expression also correlate responders to ICI with programs for T-cell activation and interferon signalling. Whilst these studies correlate responders to patients with an activated immune response, it is by no means a discriminatory marker and does not provide rational targets for resistance mechanisms.

Advances in technology have allowed the characterisation of tumoural microenvironments beyond the T-cell and myeloid compartments, using techniques such as single-cell phenotyping or multi-spectral immunohistochemistry, which can characterise over 50 proteins simultaneously (70). More recent studies go beyond phenotyping alone and explore resistance mechanisms using high-throughput transcriptomic analyses of over 18,000 genes, identifying two genes associated with ICI resistance in NSCLC (*RPL13A* and *GNL3*). Insights from analytic platforms such as these identify novel biomarkers as potential targets for combination treatment (71) (**Table 2**).

**Table 2:** Proposed biomarkers of immunotherapy resistance.

Tumour biomarkers		TME biomarkers	
PD-L1 expression	• Low or absent	Immunosuppressive cells	• Increased Tregs, MDSCs, TAMs
Tumour mutational burden	• High	Immunosuppressive cytokines/chemokines	• Increased IL-6, IL-8, CXCL10
Tumour neoantigens	• Loss or downregulation	Effector immune cells	• Decreased TILs, e.g., CD8+ T, CD4+ T, B, NK, dendritic cells
Epigenetic signatures	• DNA methylation of genes encoding immune checkpoints, antigen presentation machinery, angiogenesis	Tertiary lymphoid structures	• Decreased quantity, size, maturity
Genomic alterations	• *EGFR* mutations • *ALK* rearrangements • *STK11* mutations • *KEAP1* mutations	Abnormal signalling pathways	• Decreased IFN/JAK/STAT signalling • Increased signalling of Wnt/*β*-catenin, PI3K/AKT/mTOR, MAPK pathways
		Transcriptomic signatures	• Increased expression of genes involved in mesenchymal transition, immunosuppression, and angiogenesis
Circulatory biomarkers		Host biomarkers	
ctDNA	• Present/increased at baseline • Stable/rising on treatment	Microbiome	• Reduced microbial diversity of digestive/respiratory tract • GI tract microbiome enriched, e.g., *Bacteroidales* species • Respiratory tract microbiome enriched, e.g., *Fusobacterium nucleatum*, *Haemophilus influenzae*, *Neisseria perflava*
CTCs	• Present/increased		• Recent antibiotics
Exosomes	• Increased exosomal PD-L1 expression • Particular micro-RNA signatures		
Soluble proteins	• Elevated inflammatory cytokines/chemokines • Elevated CRP or LDH		
Peripheral immune cells	• Increased circulating Tregs • Increased NLR • Decreased CD8+/CD4+ T cells • Decreased T-cell receptor repertoire		

### Tumour microenvironment

The tumour microenvironment (TME) refers to the complex and dynamic ecosystem in which a tumour resides. It comprises stromal and immune cells, signalling molecules, structural support from the extracellular matrix, and surrounding vasculature (24). The cellular composition, signalling pathways, and metabolic conditions within the TME play a critical role in promoting or inhibiting tumour cell growth. The resulting degree of “immunogenicity” significantly influences the host response to anti-cancer therapies. Consequently, there is increasing research interest in identifying both cellular and metabolic TME biomarkers, as well as exploring how these could be clinically utilised to predict immunotherapy response or targeted to overcome immunotherapy resistance.

Numerous immune cells infiltrate the TME, with the presence and balance of specific cell types and their associated chemokines either driving progression or inhibiting malignant growth and development (24). Broadly speaking, the immune infiltrate comprises “effector” and “regulatory” cellular components. Effector cells are primarily responsible for eliminating malignant tumour cells, whereas regulator cells promote immune tolerance and evasion (50). Early TME analyses in melanoma defined three distinct TME phenotypes: those with a high T-cell prevalence within the tumour core (T-cell predominant or T-cell inflamed), those with T-cell presence largely confined to the stromal banks at the tumour periphery (T-cell excluded), and those containing few—if any—activated T cells (T-cell poor or non-T-cell inflamed) (72). Broadly speaking, the T-cell-inflamed, immune-active TME phenotype enhances anti-tumour immunity and responds well to ICI mono- or combination therapy (73). The effector T-cell poor, immune-suppressive TME however, is more likely to demonstrate evidence of chronic inflammation and display immunotherapy resistance (50).

One of the most studied biomarkers within the TME is activated effector T cells, or “tumour-infiltrating Lymphocytes” (TILs). The density, location, and proximity of TILs to cancer cells within the TME have both prognostic and predictive value in NSCLC. An abundance of TILs—especially cytotoxic CD8+ T cells—has been associated with improved clinical outcomes and sensitivity to ICI blockade in various solid tumours, including NSCLC. In contrast, a TME lacking T-cell infiltration appears to confer relative resistance (74–76). In the case of cytotoxic T cells, evaluation of their activation status has been proposed as an additional marker to improve predictive potency. Co-expression of PD-L1 or CD39 on CD8+ T cells, for example, has been associated with increased PD-1 axis blockade and improved survival in NSCLC (77, 78). Furthermore, TILs may represent a more accurate biomarker than TMB in their prediction of ICI response in the PD-L1 negative population (79, 80).

Beyond CD8+ and CD4+ T cells, additional TILs—including B cells, natural killer (NK) cells, and mature dendritic cells—have been able to differentiate between immunotherapy responders and non-responders (4). An abundance of intra-tumoural B cells was able to predict anti-PD-L1 efficacy in NSCLC in a 2022 study by Patil et al.—and a greater presence of B and NK cells within the TME was able to predict clinical benefit, as well as response durability with pembrolizumab in NSCLC (81, 82).

Tertiary lymphoid structures (TLS) also appear to hold positive predictive and prognostic value in several tumour types, including NSCLC. TLS are ectopic lymphoid formations that develop within non-lymphoid structures—typically at sites of chronic inflammation, infection, or tumour. These structures are thought to facilitate local immune responses by providing an environment where antigens can be presented and adaptive immune responses can be initiated or maintained. Recent work by Weng and colleagues reported improved survival outcomes in NSCLC patients whose tissue samples contained TLS compared to those that did not. They also reported a significant association between TLS quantity and size and ICI efficacy (83). Furthermore, TLS maturity has been linked to improved ICI response and survival (84), as well as rates of major pathological response (MPR) in the neoadjuvant treatment of NSCLC (85).

In contrast to the above-discussed TME effector cells as favourable predictive biomarkers, numerous regulatory immune cells have been associated with ICI resistance. Regulatory T-cells (Tregs)—a subpopulation of CD4+ T cells—act to inhibit the activation, proliferation, and survival of effector T cells through the production of immunosuppressive molecules, e.g., transforming growth factor-α (TGF-α) and interleukin-10 (IL-10). This results in inhibited anti-tumour immunity and the promotion of tumour immune evasion (15, 23). Increased presence of Tregs within the TME can predict resistance to immune checkpoint blockade, reduced survival, and risk of relapse in NSCLC (86, 87). Myeloid-derived suppressor cells (MDSCs) are immature myeloid cells that display a number of immunosuppressive functions—including inhibition of T-cell function, promotion of angiogenesis and T-cell apoptosis, and differentiation of effector T cells to regulatory T cells (15, 34, 50). Accumulation of MDSCs has been associated with reduced immunotherapy response, disease progression, and recurrence (23). Tumour-associated macrophages (TAMs), in particular the M2 “immunosuppressive” phenotype, suppress anti-tumour immunity and recruit other immunosuppressive cells to the TME. These have also been implicated in poor prognosis and ICI resistance in NSCLC (29, 50, 51). Pre-clinical studies targeting and modulating TAMs—for example, utilising chemokine chemokine ligand 2 (CCL2) or toll-like receptor 8—have shown synergistic results when administered in combination with ICB therapy (23).

Particular cell types within the TME have been quantified using a variety of methods, including IHC, flow cytometry, and single-cell RNA sequencing. Emerging technologies such as multiplex immunohistochemistry/immunofluorescence (mIHC/mIF) will allow the study of multiple immune cell subtypes, immune checkpoints, and functional proteins simultaneously.

Although many of these proposed TME biomarkers hold considerable promise, several limitations exist. Whilst several of these makers can be assessed through straightforward haematoxylin and eosin (H&E) staining (which would appear relatively easy to incorporate into clinical practice), TME analysis classically involves obtaining tumour tissue via an invasive procedure of some form—highlighting the need for surrogate markers that can be collected less invasively. Secondly, studies evaluating the predictive value of these TME biomarkers—for example, CD8+ T cells—have utilised various cut-off values and employed disparate scoring systems, limiting the comparability of TME analysis in clinical practice (50). Furthermore, the predictive value of TILs may be diminished in patients expressing markers of T-cell exhaustion (23, 29, 88).

### Gene expression profiling and transcriptomic signatures

To analyse the numerous cell types within the TME, along with their activation status and signalling pathway activity, a number of transcriptomic signatures (i.e., patterns of gene expression) have been developed. These are based on mRNA data from targeted or whole transcriptome RNA sequencing and vary in the number of genes assessed and platforms utilised (24, 34). Several of these signatures have been shown to have predictive value in forecasting immunotherapy response in NSCLC (34, 89).

Transcriptomic signatures enriched for genes involved in T-cell activation and cytolytic activity, interferon signalling, and DNA damage response have been able to predict improved response and survival outcomes with immunotherapy (34, 90–92). Conversely, the transcriptomes of ICI non-responders have been found to upregulate genes involved in mesenchymal transition, immunosuppression, and angiogenesis (93, 94). Jang and colleagues developed a 59-gene expression profile signature, which classifies tumours as having a “good” or “bad” tumour immune microenvironment (TiME)—predicting an increased likelihood of immunotherapy response or resistance, respectively (95). Whilst these bioinformatic techniques demonstrate considerable promise, they require sophisticated instrumentation, analysis, and interpretation—meaning they are most widely utilised in the research setting at present. Further significant validation is required prior to their incorporation into routine clinical use.

Although the complexities of the cellular, metabolic, and signalling interactions within the TME are not yet fully understood, further insights in this field are revealing strategies to target not only cancer cells but also the supportive environment that sustains and promotes their survival. The development of comprehensive transcriptomic profiling strategies is elevating this area of biomarker research—and is increasingly being used in combination with other biomarkers to provide composite prediction models that may provide more accurate forecasting of immunotherapy response.

### Tumour neoantigens

Neoantigens can be predicted based on their MHC-I binding affinity, HLA typing, and the inference of neopeptides (34, 50). The quantity and quality of neoantigen response may be able to predict immunotherapy resistance, with downregulation of neoantigens or loss of intratumour heterogeneity (ITH) representing mechanisms of immune evasion and resulting ICI resistance (3, 18, 96). Downregulation of key neoantigens was predictive of resistance to anti-PD-1 and anti-CTLA-4 in biopsies of relapsed NSCLC (18). A 2018 study by Gettinger et al. reported a positive correlation between predicted MHC-I neoantigen burden and ICI response rate and durability in NSCLC (97). Additionally, work by McGranahan and colleagues suggests that the combination of high TMB status and low neoantigen ITH may have a stronger ability to predict ICI response in NSCLC than TMB alone (98).

Advances in NGS and other biotechnologies, enabling rapid tumour sequencing, HLA-typing, and characterisation of tumour neoantigens in a high-throughput manner, have been crucial to the development of combination anti-tumour therapies—such as personalised vaccines and HLA-restricted cellular therapies (34, 99). However, there is currently no standardised platform for neoantigen identification, with existing methods relying on computational prediction and bioinformatic pipelines.

### Epigenetic signatures

Epigenetic signatures refer to potentially heritable changes in gene expression that do not alter the underlying DNA sequence but can significantly impact tumour behaviour and treatment response. They play an important role in both carcinogenesis and anti-tumour immunity, mediating factors such as antigen-presenting MHC molecules, immune checkpoint molecules, neoantigen processing, and lymphocyte activation (3, 34). Emerging evidence suggests that epigenetic signatures can mediate immunotherapy resistance, positioning them as potential predictive biomarkers.

A key mechanism of epigenetic regulation is DNA methylation, which typically suppresses of gene expression. Hypermethylation of promoter regions in genes encoding immune checkpoints or antigen presentation machinery can facilitate immune system evasion and ICI resistance (15, 16). For instance, the “EPIMMUNE” epigenomic profile, for example—a microarray DNA methylation signature—has been associated with ICI response and survival in NSCLC. A multi-centre study of 142 patients with stage IV NSCLC previously treated with anti-PD-1 therapy reported the EPIMMUNE-positive predicted both ICI response and improved survival outcomes. Notably, it was not associated with PD-L1 expression, CD8+ cell presence, or mutational load. The EPIMMUNE signature was further validated in two independent patient cohorts (100). Conversely, the absence or decreased levels of lysine-specific demethylase 1A (LSD1), along with increased PD-L1 K162 methylation and SET domain-containing lysine methyltransferase 7 (*SETD7*) expression, have been associated with anti-PD-L1 resistance in NSCLC (101).

Integrating epigenetic signatures into clinical practice also involves the use of high-throughput technologies, such as NGS and epigenomic profiling. Advancing our understanding of epigenetic alterations could facilitate the development and utilisation of epigenetic therapies, which represent a promising avenue by which to enhance the efficacy of existing ICI therapies, with encouraging anti-cancer activity observed in pre-clinical studies (102, 103).

### Genomic alterations

Genomic alterations in particular tumour suppressors or oncogenes have also been shown to influence NSCLC sensitivity to immunotherapy—often through modulation of TME immunogenicity. With nearly 50% of NSCLC cases harbouring potentially targetable driver gene changes, the ability of specific somatic mutations to predict immunotherapy response has been subject to considerable research interest.

#### Negative predictors

Several somatic mutations implicated in oncogene-addicted NSCLC are associated with limited response or primary resistance to immune checkpoint blockade. These cancers typically exhibit improved responses with tyrosine kinase inhibitors (TKI) that specifically target these mutations or their resulting aberrant signalling pathways.

#### *EGFR* and *ALK/ROS1* rearrangements

Activating mutations in the epidermal growth factor receptor (*EGFR*) gene are found in approximately 10%–30% of NSCLC cases, and up to 60% in Asian populations (104, 105). This mutation is associated with downstream signalling pathways, including MAPK/ERK, PI3K/AKT, and BAX/BCL-2. The phenotype for these mutations is typically that of younger, never-smokers. In non-smokers, resistance to immunotherapy has been related to the development of multiple sub-clones, increased infiltration of immunosuppressive cytokines, Tregs and TAMs (106), reduced CD8+ T-cell infiltration, reduced TMB/neoantigen load, and a reduced interferon-gamma signature—all culminating in an inert tumour microenvironment (24, 107).

The clinical benefit of PD-1/PD-L1 inhibition in this *EGFR*-mutant population is generally poor (3, 104)—although some data suggest that this may not apply to the exon 20 insertion subgroup (108, 109). A phase II study by Lisberg and colleagues—designed following observations made in the KEYNOTE-001 trial—found the efficacy of first-line anti-PD-1 therapy in an *EGFR*-mutant, PD-L1+ population to be minimal (almost zero) (110). The IMpower150 study noted no benefit from atezolizumab and bevacizumab combined with chemotherapy in advanced *EGFR*-mutant NSCLC (111). A 2017 meta-analysis of *EGFR*-mutant NSCLC similarly reported that ICI did not improve OS over chemotherapy when administered in the second-line setting (112). Similar findings were described in KEYNOTE-010, CheckMate057, and OAK (9, 36, 48).

There is conflicting data regarding the impact of *EGFR* status on PD-L1 expression levels—with some analyses suggesting that PD-L1 expression may be inherently reduced in EGFR-mutant tumours, whilst others describe no correlation, or even the opposite effect (7, 107, 113, 114).

Rearrangements in the anaplastic lymphoma kinase (*ALK*) gene have also been associated with relative resistance to immune checkpoint inhibition. Several clinical trials and retrospective studies have demonstrated the utility of *ALK* rearrangement as a negative predictor for anti-PD-1/PD-L1 response (9, 36, 48, 111). For instance, the ImmunoTarget study reported no response (ORR 0%) to ICI monotherapy in an *ALK*-rearranged cohort (115), and similar results were observed with durvalumab therapy in the phase II ATLANTIC trial (116). Other non-smoking NSCLC phenotypes associated with reduced ICI sensitivity include *ROS1* rearrangements and mutations in *HER2* and *BRAF*.

#### *STK11* mutations

Serine/threonine kinase 11 (*STK11*) is a tumour suppressor gene that encodes the liver kinase B1 protein (LKB1). Through direct phosphorylation and activation of AMP-activated protein kinase (AMPK), *STK11* plays a several roles in cancer cell cycle regulation, differentiation, metabolism, angiogenesis, and the DNA damage response (104). Loss of *STK11* function or mutations in *LKB1* have been identified in 10%–20% of NSCLC and are believed to confer inferior survival outcomes and relative ICI resistance in advanced NSCLC when compared to wild-type tumours (117, 118). The underlying mechanism has been linked to the *STK11* genetic alteration, resulting in an indolent, immunosuppressive tumour milieu, characterised by lower infiltration of TILs, reduced expression of inflammatory cytokines, and epigenetic inhibition of the stimulator of IFN genes (STING). Increased expression of PD-L1 despite a moderate to high TMB has also been described (3, 104).

A sub-analysis of 47 patients within the phase II LUNG-MAP study, evaluating talazoparib (PARP inhibitor) and avelumab (anti-PD-L1) in advanced non-squamous NSCLC with pathogenic *STK11* genomic alterations did not meet pre-specified efficacy thresholds (ORR 2%). However, durable disease stabilisation was observed, with one patient remaining on treatment for more than 14 months (119). Other studies targeting this challenging *STK11*-low tumour group are currently recruiting.

#### *KEAP1* mutations

Kelch-like ECH-associated protein 1 (*KEAP1*) functions as an adaptor protein for Cullin 3, negatively regulating NRF2 activity and influencing the oxidative damage response. Mutations in the *KEAP1* gene have been found in 12%–19% of NSCLC and appear to result in lower infiltration of CD8+ and natural killer cells (3, 104). *KEAP1* mutations are thought to predict primary ICI resistance and inferior prognosis compared to wild-type tumours, regardless of the therapy used (120, 121).

#### Positive predictors

In contrast to the somatic mutations discussed above, which appear to confer immunotherapy resistance, a number of genetic alterations with potential positive predictive value have also been identified.

#### TP53

The tumour suppressor gene *TP53* has been associated with a poorer prognosis in lung cancer, with mutations in this gene identified in more than 50% of cases. Loss of *TP53* function has been associated with high tumour mutational burden, increased CD8+ T-cell infiltration, and increased PD-L1 expression (in the case of *TP53* missense mutations) (3, 104). Together, these factors may facilitate an inflamed TME phenotype—and this mutation has been correlated with improved response rates and survival in advanced NSCLC treated with ICI (117, 122).

#### Mismatch repair deficiency

Another promising biomarker for predicting immunotherapy response in NSCLC lies in the mismatch repair (MMR) pathway. It is estimated that 4%–5% of NSCLC have MMR alterations—with the predictive value of MMR genes *MLH1*, *MSH2*, *MSH6*, and *PMS2* already well recognised in GI malignancy. Studies are currently evaluating the impact of dMMR status in NSCLC immunotherapy response and survival (e.g., NCT02983578). Data thus far have largely supported MMR as a positive predictor of immunotherapy response, and pembrolizumab is now FDA-approved for use in dMMR-positive solid tumours that are metastatic or unresectable in patients without alternative therapeutic options (123).

#### KRAS

Oncogenic mutations in *KRAS* are seen in approximately 20%–30% of NSCLC and are particularly prominent in smokers with adenocarcinoma (104). *KRAS*-mutated tumours typically demonstrate an immunosuppressive TME, with secretion of immunosuppressive inflammatory cytokines, infiltration of immunosuppressive immune cells, and higher levels of PD-L1 expression (124). Despite their classically “non-inflamed” TME phenotype, studies suggest that *KRAS* mutations alone confer sensitivity to immunotherapy—although the data on this are variable. For example, patients with *KRAS* mutations in a CheckMate-057 subanalysis saw superior benefits with nivolumab compared to the *KRAS* wild-type cohort (48). These findings are further supported by outcomes from the BIRCH trial and a 2017 meta-analysis by Kim et al. (125, 126).

Interestingly, *KRAS-*mutated tumours may demonstrate differing biology and therapeutic vulnerabilities depending on the particular mutation subtype, as well as co-occurring genetic alterations. In a 2019 study by Jeanson and colleagues, NSCLC patients with G12D, G12V, and G13C *KRAS* mutations exhibited higher PD-L1 expression compared to patients with G12A or G12C *KRAS* mutations—although the clinical outcomes with immunotherapy were not significantly different between the groups (127). In contrast, a 2022 study by Liu et al. concluded that the G12D *KRAS* mutation subtype appears to drive primary resistance to anti-PD-1/PD-L1 therapy (128). The impact of *KRAS* co-mutation also appears predictive of immunotherapy response—with *KRAS/TP53* co-mutation typically predicting sensitivity to ICI, whilst *KRAS/STK11* or *KRAS/KEAP1* co-mutations appear to predict resistance (129–131). The resistance conferred by *KRAS/STK11* co-mutation is believed to be more marked than *STK11* mutation in isolation and is associated with a particularly aggressive disease phenotype (132). Conversely, a 2017 study by Dong et al. saw improved survival with pembrolizumab in both the *KRAS* and *TP53* single mutation subgroups—with a more remarkable clinical benefit in tumours with *KRAS/TP53* co-mutation (133).

### Microbiome

Another emerging biomarker receiving significant attention in recent years is the host microbiome. This refers to a dynamic collection of commensal micro-organisms colonising a particular environment and is believed to impact anti-cancer immunity as well as the efficacy and toxicity of immunotherapy (3, 34). The microbiome can modulate the immune response by modifying T-cell function, regulating cytokine production, and influencing the balance between regulatory and effector T cells (50).

The most studied thus far has been the microbiome of the host digestive tract. Several pre-clinical studies in mice have highlighted the role of the gut microbiome in solid tumour response to immunotherapy (134–136), and a landmark 2018 study by Routy et al. demonstrated that NSCLC patients with a more diverse gut microbiome—particularly those enriched for *Akkermansia muciniphila* and *Enterococcus hirae*—saw improved disease response to PD-1 inhibition. Furthermore, those who received antibiotics known to affect gut microbiota had decreased survival when these were given within 3 months of PD-1 blockade) (137). Following this, multiple other studies have reported a significant correlation between lower bacterial diversity and a lack of enrichment of particular micro-organisms, with ICI resistance (138, 139). Non-responders may be more likely to be enriched for *Bacteroidales* species, for example, whereas responders may have an increased representation of *Faecalibacterium* and *Ruminococcaceae* (136, 140, 141).

There is also emerging evidence to support the respiratory tract microbiome as a potential biomarker for immunotherapy resistance in NSCLC (142). Patients progressing whilst receiving ICI therapy were found to have reduced respiratory microbiome diversity in a 2022 study by Masuhiro et al. In addition, enrichment for specific micro-organisms has been documented in patients with poor immunotherapy response—including *Fusobacterium nucleatum*, *Haemophilus influenza*, and *Neisseria perflava* (143, 144).

To date, analyses in this area have utilised 16S ribosomal RNA gene sequencing, metagenomics shotgun sequencing and quantitative polymerase chain reaction (PCR) to describe the microbiome signature. The optimal method of analysis, however, is yet to be ascertained (34, 145). An influential study by Poore and colleagues, for example—originally published in 2020 and subsequently retracted due to concerns regarding the robustness of specific microbial signatures—highlights some of the challenges of isolating the microbiome for analysis (146).

As mentioned above, exposure to antibiotics during ICI therapy has been associated with inferior clinical outcomes in NSCLC in a number of retrospective studies (137, 139, 147). It is yet to be fully determined whether this represents another independent biomarker—or rather acts as a surrogate for overall microbiome health, or patient factors such as frailty, comorbidities, and advanced age (3).

Ultimately, although the host microbiome as a biomarker for immunotherapy is largely in the pre-clinical phase at present, these insights suggest that it could be incorporated into predictive models to guide personalised treatment strategies. Furthermore, given the potential immune-modulating effects, it would seem that pausing to consider the potential implications of antibiotic use in the period prior to or during immunotherapy may be worthwhile, if possible (148). Methods to restore the diversity of gut microbiota—including dietary modification, probiotic use, and faecal transplantation—require further investigation as potential adjuncts to immunotherapy (145).

## Future biomarkers

### Circulatory biomarkers

Tissue biopsy has long been considered the gold standard in the diagnosis and evaluation of malignancy. However, availability and accessibility limit the ease with which tissue biopsy can be utilised—particularly in longitudinal monitoring of tumour biology and treatment response. Tissue biopsy is limited by sampling heterogeneity, meaning that analysis of PD-L1 expression, TMB, and TME, for example, may vary depending on specific biopsy location (34). Furthermore, patients for whom tissue biopsy is not possible or clinically inappropriate may be unfairly excluded from consideration of systemic anti-cancer therapies. Given these limitations of tissue biopsy, there has been considerable research interest in the identification and clinical application of liquid biopsy. Peripheral blood collection is comparatively non-invasive, technically easier, and less expensive to obtain, and may be able to overcome some of the heterogeneity seen with tissue sampling.

Circulating biomarkers are molecules found within the peripheral bloodstream. These include cells, proteins, nucleic acids, extracellular vesicles, and metabolites. There is a growing body of evidence supporting the use of circulating biomarkers in the prediction of response/resistance to immunotherapy in NSCLC, as well as their ability to facilitate dynamic assessment of response over time. In addition to the limitations associated with physically obtaining tissue samples, the performance of tissue biomarkers in predicting ICI resistance is known to be imperfect—with some low PD-L1 expressing and low TMB tumours still benefiting with immunotherapy treatment (9, 149). This highlights the need for additional biomarker identification with more robust predictive capabilities.

#### Circulating tumour DNA

Circulating tumour DNA (ctDNA) refers to short fragments of tumour-cell DNA, released into the bloodstream from primary tumours or metastases when a cancer cell undergoes division, apoptosis, or necrosis. Comprising a small component of cell-free DNA (cfDNA), ctDNA can be distinguished by its shorter fragment size (typically 50–150 bp) and the presence of identifiable genetic alterations associated with the underlying cancer (150, 151). Mutant sequences within ctDNA can be detected, quantified, and analysed with relative sensitivity and specificity utilising a number of different techniques, including NGS, quantitative PCR (qPCR), and droplet digital PCR (ddPCR) (152).

ctDNA appears to have both prognostic and predictive biomarker potential in NSCLC patients receiving ICI. Higher levels of ctDNA have been linked to a greater volume of underlying disease and inferior survival (62, 153). A recent study by Jun and colleagues reported inferior clinical outcomes in NSCLC patients receiving consolidation immunotherapy, where ctDNA was detectable prior to, during, and following 6 months of nivolumab +/− ipilimumab (154). Falling ctDNA levels during immunotherapy, and the degree of this change, also appear to be important. A 2021 meta-analysis of over 1,000 NSCLC patients saw no significant association between pre-treatment ctDNA and ICI response; however, declining ctDNA levels following ICI treatment were strongly associated with improved ORR, PFS, and OS (155). Furthermore, a prospective 2017 study reported superior ICI responses, PFS, and OS in NSCLC patients whose ctDNA levels became undetectable after 8 weeks of immunotherapy (156). Numerous other studies have supported this correlation—with continued presence of ctDNA on treatment, or lesser degrees of ctDNA reduction, typically conferring ICI resistance and inferior survival outcomes (157–160). Additionally, there is some evidence to support the ability of ctDNA to predict durable ICI response (161) and pathological complete response (pCR) when ICI is utilised in the neoadjuvant setting (162).

ctDNA can be utilised to identify driver mutations, gene amplifications, and epigenetic changes that are associated with immunotherapy resistance, for example, *PTEN* or *STK11* mutations (163). Via sequencing analysis, ctDNA can also be utilised as a surrogate marker for TMB (known as blood TMB [bTMB] as opposed to tissue-derived TMB [tTMB] in this instance)—with lower bTMB typically correlating with reduced immunotherapy response and inferior survival outcomes (164). bTMB has demonstrated predictive value in both first- (65, 165) and second-line (9, 91) immunotherapy treatment. The predictive cut-offs, however, remain controversial, with values ≥ 16 mut/Mb or ≥ 20 mut/Mb demonstrating predictive value in some studies—and not in others (166, 167). It is worth noting also that much of the data in this area are retrospective or exploratory. Thus far, the US FDA has approved two blood-based NGS assays with the ability to evaluate ctDNA for the presence of targetable driver mutations, bTMB and MSI in NSCLC (FoundationOne Liquid CDx and Guardant360). However, these are approved as companion diagnostics to guide targeted TKI selection in the presence of *EGFR/ALK/MET* mutations—and not for the prediction of response to immunotherapy (150).

Overall, whilst ctDNA shows considerable biomarker promise, its translation into widespread clinical use is currently limited by small study sizes, the lack of unifying detection thresholds across multiple testing platforms, and the low volume of ctDNA within plasma (62).

#### Circulating tumour cells

Circulating tumour cells are shed from primary tumour or metastatic sites, either as single cells or clusters. As with ctDNA, circulating tumour cells (CTCs) are present in very low numbers within the peripheral bloodstream, with an estimated prevalence of 1–10 CTCs/10 mL of blood (150). The quantity and molecular characteristics of CTCs can provide important prognostic information and identify potential mechanisms of resistance. The level of evidence supporting the use of CTCs in NSCLC is variable, although there are data to support their utility as a prognostic and predictive biomarker for immunotherapy. Higher CTC quantity has been associated with inferior survival outcomes and increased likelihood of disease progression, both prior to and during ICI therapy (168, 169). There is also evidence that changes in the number and phenotype of CTCs during ICI therapy may be indicative of emerging resistance (170, 171).

Additionally, a correlation between CTC PD-L1 expression and ICI response has been explored—with a number of studies noting a link between the presence of PD-L1+ CTCs at baseline, as well as increasing levels of PD-L1+ CTCs during treatment and immunotherapy resistance (172, 173). Interestingly, a 2018 paper by Guibert and colleagues reported an increased frequency of PD-L1 positivity on CTCs in comparison to tissue samples (83% vs. 41%) in a population of advanced NSCLC (168). A study by Ilie et al., however, demonstrated concordant expression of PD-L1 on paired CTC and tissue samples, and no correlation between PD-L1 CTC expression and prognosis (174). These opposing results may partly relate to the above-described lack of standardised PD-L1 testing methods.

Furthermore, owing to the ability of CTCs to provide more intact intracellular material for analysis (compared to fragmented ctDNA), CTCs can also provide transcriptomic, genomic, and proteomic data (62). Quantification of CTC indoleamine-2,3-dioxygenase (IDO) levels, for example, has been correlated with inferior immunotherapy survival outcomes (169).

#### Extracellular vesicles and exosomes

Exosomes are small, membrane-bound extracellular vesicles that mediate intercellular communication by transferring proteins, lipids, and nucleic acids. Tumour-derived exosomes are excreted into the extracellular space and bloodstream, where they can modulate the immune microenvironment. Analysing the cargo of exosomes, including PD-L1 expression and immunosuppressive microRNAs, can provide insights into tumour biology and mechanisms of resistance.

Surface exosomal PD-L1 (exoPD-L1) expression offers an alternative method for measuring PD-L1 levels. Higher exoPD-L1 expression at baseline has been associated with immunotherapy resistance and inferior survival outcomes in NSCLC populations (175, 176). The magnitude of change in exoPD-L1 following immunotherapy also appears to correlate with outcomes—with more significant increases in exoPD-L1 expression appearing to result in improved response rates, PFS and OS (177). Opposing results were seen in a study by de Miguel-Perez (178), however, potentially reflecting differing exosome isolation and analysis methods. There is conflicting data, too, regarding the concordance of exoPD-L1 and tissue PD-L1 expression—which may relate to the fact that exosome excretion is not exclusive to tumour cells, and can be secreted into the bloodstream by any cell type (150).

Studies of exosomal miRNAs have also revealed potential value in predicting immunotherapy response. A study by Peng and colleagues identified three exosomal miRNAs whose upregulation prior to treatment was associated with immunotherapy resistance in advanced NSCLC (179). Other research groups have also described particular miRNAs with the ability to independently predict inferior clinical outcomes (180, 181). PD-L1 expression on miRNAs has also been evaluated in NSCLC patients receiving immunotherapy. Higher baseline PD-L1 miRNA copies and greater dynamic changes in ICI therapy have been associated with improved immunotherapy response (177, 182).

#### Soluble proteins

A number of soluble proteins present in the peripheral circulation have also demonstrated prognostic and predictive value as potential biomarkers. For example, there has been considerable research interest in the relationship between inflammatory cytokines and chemokines and ICI response. Elevated pre-treatment levels, and stable or increasing levels of therapy, of IL-6, IL-8, and CXCL10 have been associated with immunotherapy resistance, less durable immunotherapy responses, and inferior survival outcomes (183–185).

Soluble PD-L1 (sPD-L1) levels could also hold prognostic and predictive value. A 2022 meta-analysis concluded that elevated pre-treatment sPD-L1 predicted inferior PFS and OS in lung cancer treated with ICI (185, 186). This finding has been corroborated elsewhere and has been linked with reduced TME infiltration of TILs as an explanatory mechanism (187). Results from studies evaluating the impact of sPD-L1 changes in response to immunotherapy and the correlation between sPD-L1 and tissue PD-L1 expression are mixed (188, 189).

Elevated levels of other circulatory proteins, such as C-reactive protein (CRP) and lactate dehydrogenase (LDH), have also been correlated with inferior ICI response and poor survival outcomes in NSCLC populations (190).

#### Peripheral immune cells

Further to the predictive biomarkers identified via TME analyses, studies have evaluated the importance of circulating immune cells in dictating immunotherapy response. There is evidence to suggest that higher baseline circulation of CD8+ and CD4+ T lymphocytes—as well as lower levels of Tregs—are seen in immunotherapy responders (150, 191).

The absolute neutrophil count to absolute lymphocyte count within peripheral blood is referred to as the neutrophil/lymphocyte ratio (NLR). A 2020 meta-analysis by Jin and colleagues demonstrated a correlation between high baseline NLR and shorter PFS and OS in NSCLC treated with ICI (192). Several studies have also demonstrated that high NLR are predictive of poor immunotherapy response, although the NLR cut-off to differentiate responders from non-responders is controversial (4, 50, 51, 193, 194).

TCR repertoire—referring to the diversity and clonality of T-cell receptors present within an individual’s immune system—is determinable via NGS techniques. A broad pre-treatment TCR repertoire, along with an increase in diversity during treatment, has been associated with superior ICI responses and longer PFS (195).

Circulating biomarkers represent a valuable tool in predicting response to immunotherapy in NSCLC, with liquid biopsy allowing a minimally invasive means of repeated sampling for longitudinal disease monitoring. There is potential for circulating biomarkers to detect emerging resistance, offering insights into the evolving tumour genome and facilitating timely adjustments in treatment strategy. This ability to track cancer in real time also enables early detection of recurrence after curative treatment or resistance to ICI in the metastatic setting. Although the evidence supporting many of the above-discussed biomarkers is mounting, many are not yet ready for routine clinical application. Future research should focus on validating these biomarkers in large, prospective clinical trials and developing standardised assays and optimal cut-offs for their measurement.

### Radiomics

Radiomics—an emerging field of medical imaging that involves the extraction of large volumes of quantitative data to support clinical decision-making—is also demonstrating considerable promise in the prediction of immunotherapy resistance in NSCLC. For example, PD-L1 imaging, utilising PET tracers to identify tumours more likely to respond to ICI therapy, is an area of ongoing research interest. A detailed discussion of radiomics, however, is beyond the scope of this review.

## Novel strategies to overcome resistance

Immunotherapy resistance remains one of the most challenging areas of unmet need in oncology, with durable ICI response seen in only a minority of patients. In the quest to overcome immunotherapy resistance, considerable attention has been directed towards combination treatment strategies. These have involved the addition of chemotherapy, radiation, and co-stimulatory or co-inhibitory ICI to the existing anti-PD-1/anti-PD-L1 backbone. For advanced NSCLC with PD-L1 TPS > 50%, anti-PD-1, or anti-PD-L1 monotherapy is an option—with approvals for pembrolizumab, cemiplimab, or atezolizumab monotherapy. Of these three agents, pembrolizumab monotherapy has the longest follow-up, with a 5-year overall survival estimate of only 31.9%, highlighting the need to overcome resistance even in patients with high PD-L1 expression (196). The addition of chemotherapy or anti-CTLA4 to anti-PD-1 has demonstrated improvements in overall survival, even in patients with PD-L1 < 50%, in KEYNOTE-189 and CheckMate-227, respectively (6, 11). Additional combination strategies have aimed to increase neoantigen quantity (e.g., epigenetic modulation mechanisms), alter neoantigen quality (e.g., personalised neoantigen vaccines), bolster the antigen presentation process (e.g., oncolytic viruses), increase effector T-cell function (e.g., anti-GITR, anti-OX40, anti-ICOS), or inhibit immunosuppressive components within the TME (e.g., IDO, TGFβ, VEGF, PI3K) (2, 70, 197). Additional novel therapeutics, such as bispecific T-cell engagers and adoptive T-cell therapies have also been explored. However, there are no direct head-to-head studies to compare these different combinations, nor biomarkers to guide the selection of one combination over another (198). Furthermore, despite strong biological rationale and encouraging phase II studies, many of these combinations have failed due to the lack of robust predictive biomarkers to guide combination selection, e.g., TIGIT (199–201). This highlights the importance of incorporating comprehensive biomarker analyses into the selection of combination treatment regimens. A multi-omic perspective enriches clinical understanding of underlying tumour biology and has the potential to overcome some of these current challenges, optimising treatment precision and thereby therapy response rates (70).

A key step in overcoming immunotherapy resistance involves the use of novel technologies to understand the mechanisms of resistance, and thereby the identification of relevant predictive biomarkers. In turn, this facilitates the discovery of new therapeutic targets and potential treatment strategies. NGS technology, for example, has been transformative in the management of NSCLC. Its ability to provide a comprehensive snapshot of the individual genetic and molecular profile of a tumour enables the recognition of specific immune signatures that may underpin ICI resistance. In turn, this information can be utilised to select a customised precision oncology treatment strategy (202–204). The previously mentioned aberrant signalling pathways, for example, can now be studied utilising transcriptomic and epigenomic analyses. Integrating the evaluation of these signalling pathways into clinical practice could enhance immunotherapy personalisation—with specific targeting alongside ICI administration presenting a potential opportunity for synergy and restoration or maintenance of immunotherapy sensitivity. Advances in single-cell RNA sequencing (scRNA-seq) can provide insights into TCR diversity and the heterogeneity of TILS, with the potential to characterise rare cellular subpopulations that contribute to increased or decreased ICI response (197). CRISPR (clustered regularly interspaced short palindromic repeats) and gene editing technology enable the identification and manipulation of genetic material in order to enhance immunotherapy efficacy—for example, via the selective knockout of genes associated with immune evasion or ICI resistance, or via the genetic modification of T cells to potentiate their functionality (202, 205, 206). Finally, artificial intelligence (AI) and machine learning models—which provide advanced data analysis capabilities—are also demonstrating increasing promise in the identification of novel predictive biomarkers. AI algorithms have the potential to efficiently scrutinise vast datasets and identify novel biomarkers predictive of response or resistance to immunotherapy (202). By leveraging these technological advances, NSCLC treatment strategies can be further personalised, and patient outcomes improved. For these novel technologies to reach their full potential, however, testing platforms will require universal calibration standards that minimise variability and maximise the reliability of results.

A further strategy with significant potential to progress the translation of biomarker research toward routine clinical practice is to incorporate biomarkers into clinical trial design. This can be achieved by utilising a biomarker enrichment strategy—in which biomarkers are applied to select a particular study population for initial therapy (e.g., high PD-L1 expressing NSCLC, recruited to receive ICI monotherapy)—followed by customisation of the therapeutic approach as guided by disease response or lack thereof. Responders can continue the initial line of therapy, whilst those in the non-responder cohort proceed to treatment intensification (e.g., a combination regimen) guided by specific biomarkers and the suspected primary resistance mechanism. This format has the potential to elevate trial outcomes, facilitate the delivery of individualised therapy, and reduce unnecessary toxicity from ineffective treatments (197). Alternatively, a biomarker stratification design can be selected, dividing participants into disparate subgroups based on the presence or absence of a particular biomarker. Response to study treatment is then evaluated within each subgroup, demonstrating potential clinical scenarios in which biomarker-guided treatment decisions could be beneficial (197). Adjustment of therapy in this biomarker-guided way—acknowledging the complexity of immunotherapy resistance mechanisms, and the interactions between underlying tumour biology and the host immune system—will be pivotal to implementing effective combination therapeutics.

The introduction of ICI to the neoadjuvant setting provides a unique opportunity to assess biomarkers on matched samples and adapt adjuvant treatment rationally based on the mechanism of resistance. There is currently one neoadjuvant study (CHECKMATE-816) (162) and five perioperative ICI studies in this space (AEGEAN, CHECKMATE-77T, KEYNOTE-671, NADIM II, NEOTORCH) (207–211), with many more ongoing. Studies such as NEOSTAR (nivolumab and chemotherapy ± ipilimumab) and neoadjuvant nivolumab and relatlimab (anti-LAG3) are good examples of the potential that a neoadjuvant platform offers in which to study biomarkers of immunotherapy response using multi-omic techniques (212, 213). The phase II NEOSTAR trial noted improvements in major pathological response (MPR) when ipilimumab was added to a neoadjuvant chemotherapy and nivolumab regimen—from 31.1% (7/22) to 50% (11/22). Exploratory endpoints utilising single-cell sequencing and multi-platform immune profiling underscored particular immune cell populations and phenotypes that were preferentially increased in the combination ipilimumab, nivolumab, and chemotherapy cohort. Pre-treatment gut microbiota of patients achieving MPR were enriched with beneficial organisms such as *Akkermansia*. The phase II trial of pre-operative nivolumab ± relatlimab reported improved major pathological and objective radiographic responses in the nivolumab and relatlimab cohort (30% vs. 27%, and 27% vs. 10%, respectively). The combination arm also noted improved DFS and OS outcomes after a median follow-up of 12 months. Exploratory analyses of metabolic responses, immune cell phenotyping, gene expression profiling, and genomics also provided insights into biological processes triggered by pre-operative immunotherapy.

**Figure 1:**
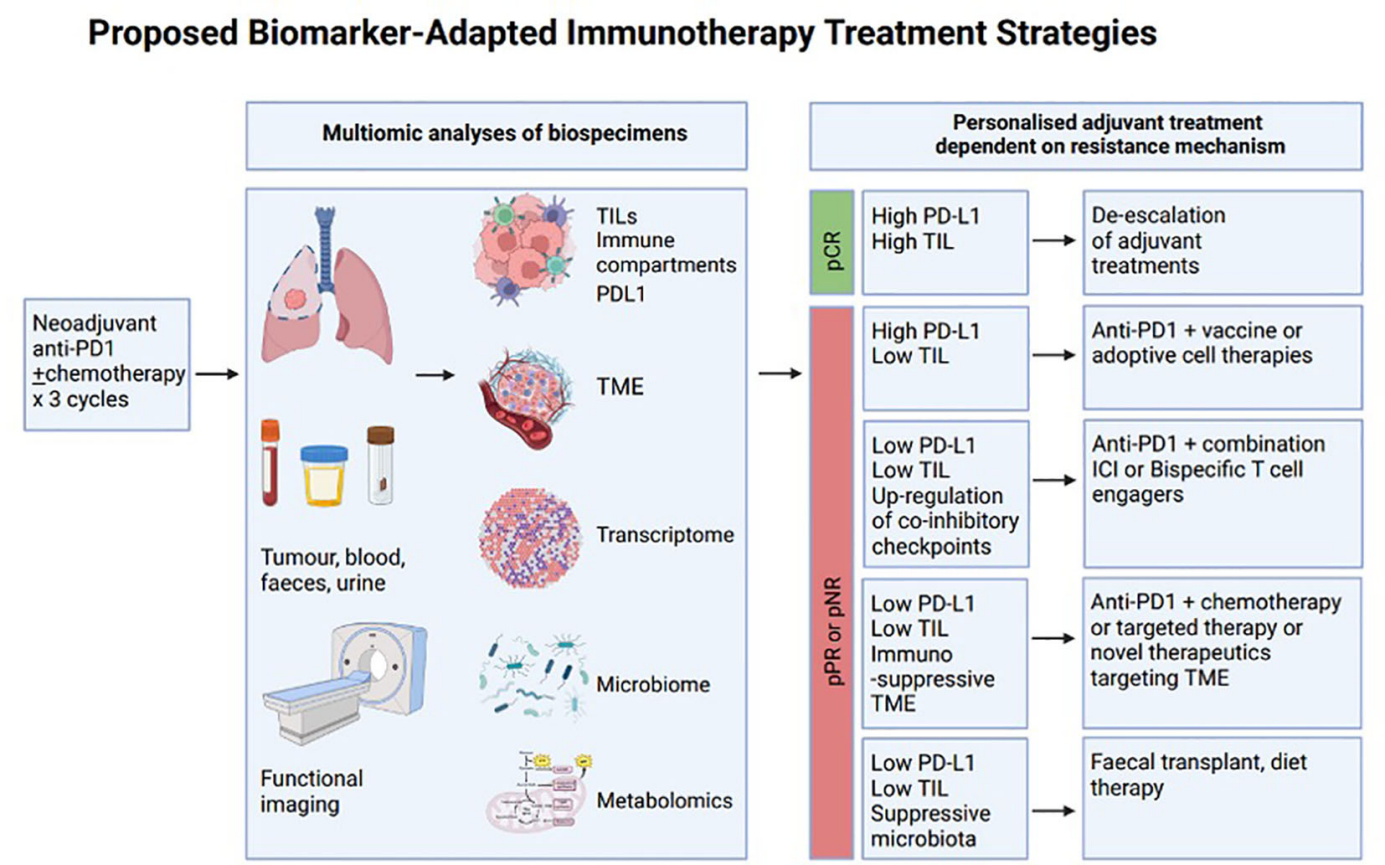
A proposed schema for biomarker-adapted immunotherapy selection. Comprehensive multi-omic biomarker analyses of various biospecimens (including tumoural tissue, blood, sputum, urine, etc.) in conjunction with functional imaging. Evaluation of matched samples prior to and following neoadjuvant therapy can characterise changes within a tumour, which can then be harnessed to guide the selection of subsequent adjuvant treatment. A pathological complete response could potentially lead to treatment de-escalation, whilst pathologic partial responders or non-responders could undergo treatment escalation guided by the primary drivers of immune resistance. The use of identified immune signatures could help identify combination therapeutics with an increased likelihood of response for each participating individual. Whilst the allocated treatments shown here are definitive, this simply serves to illustrate a potential framework whereby therapy selection can be stratified leveraging existing biomarkers. Anti-PD1, anti-programmed death-1 monoclonal antibody; ICI, immune checkpoint inhibitor; PD-L1 programmed death-ligand 1 expression; pCR, pathologic complete response; pPR, pathologic partial response; pNR, pathologic non-responder; TILs, tumour infiltrating lymphocytes; TME, tumour microenvironment.

These neoadjuvant and peri-operative studies have mandated adjuvant treatments that are not adapted according to the pathological findings from surgery. Pathologic response after neoadjuvant treatment has been shown to be an important surrogate for clinical endpoints such as event-free survival, whilst overall survival data are still awaited. Non-responders, therefore, present an excellent opportunity to study mechanisms of resistance and potentially incorporate an “umbrella”-style design to allocate adjuvant treatment based on evolving tumour biology (**Figure 1**). Examples in melanoma research have paved the way in terms of adaptive umbrella trial design (214–216). The “Lombard Street Approach”, for example, describes an innovative platform that emphasises flexibility and responsiveness in neoadjuvant trial protocols—integrating multiple biomarkers to guide sequential decision-making and personalised treatment modulation based on real-time biomarker feedback. The back-and-forth approach of characterising non-responders and identifying potentially effective alternative treatment combinations, which are then trialled in sequentially smaller subgroups of patients with unfavourable tumour characteristics, can accelerate trial efficiency and improve patient-centred care (216). These melanoma studies highlight the potential to use the neoadjuvant setting as a window-of-opportunity platform to personalise adjuvant treatment before expanding into larger combination studies. Beyond this, adaptive trial frameworks leveraging biomarker-tailored combination immunotherapy regimens have been described—for example, in glioblastoma (217). NEOCOAST, a platform study in the neoadjuvant lung cancer setting, enables the assessment of multiple drugs with the generation of rich clinical and translational data to assess response and resistance mechanisms with multiple agents (218). Insights from multi-omic biospecimen analyses can identify key resistance biomarkers and enable potential personalisation of ICI combinations in response (**Figure 1**). Such an endeavour would require well-annotated matched biospecimens before and after neoadjuvant treatment, as well as high-throughput bioinformatic pipelines with the ability to incorporate clinical and radiological parameters, as well as immunoprofiling of both the tumour microenvironment and host context.

## Clinical implications and future directions

Although immunotherapy has markedly transformed the treatment of NSCLC, resistance remains a substantial challenge. As such, the identification of reliable and accurate biomarkers with which to predict immunotherapy response is critical. The clinical implications of these biomarkers are vast. Firstly, by offering insights into both patient prognosis and suspected treatment response, biomarkers can allow contextualised clinical decision-making in NSCLC. By facilitating patient stratification to appropriate treatment regimens, therapy can be tailored not just to histological cancer type, but to a patient’s specific tumour characteristics and biology. Importantly, for those less likely to respond to immunotherapy, biomarker-driven patient selection allows for intensification of therapy or a shift to an alternative treatment approach, thereby avoiding ineffective therapy and unnecessary toxicity. In addition, biomarker research furthers our understanding of immunotherapy resistance at a molecular level, which in turn has the potential to inform novel therapy design and early-phase clinical trials. Alongside the rise of liquid biopsy for diagnostic purposes, the value of circulating biomarkers in providing both prognostic and predictive information is becoming increasingly apparent. Although their utilisation remains limited to the research and clinical trial settings at present, these circulating biomarkers hold unprecedented potential to provide non-invasive, real-time monitoring of tumour growth, treatment response, emerging resistance, and disease recurrence. This opportunity for longitudinal disease monitoring permits the dynamic adaptation of the therapeutic approach over time, further enhancing personalised oncological care. Finally, the value of prognostic and predictive biomarkers in guiding clinical decisions in early NSCLC is increasingly clear. Residual ctDNA, for example, may be able to identify those at higher risk of disease recurrence post-operatively who may benefit from a more intense treatment approach (219). Alternatively, undetectable ctDNA following definitive chemoradiation therapy could predict a group of patients with favourable clinical outcomes in whom treatment de-escalation could be considered, for example, with the omission of consolidative immunotherapy (220).

The widespread integration of NSCLC biomarkers into routine clinical practice is challenged by several factors. Notably, the predictive accuracy of many of the above-discussed biomarkers has varied considerably across studies, due to heterogeneous detection methods, definitions, and cut-off values utilised. Research aimed at standardising biomarker testing protocols and thresholds, as well as further validation and refinement of these biomarkers through large-cohort, prospective trials, is required before their incorporation into standard clinical pathways. In addition, it is essential to evaluate these biomarkers in a broad variety of clinical contexts and to gather longitudinal data with which to explore their long-term implications on patient survival and quality of life. Finally, whilst there is a large body of evidence supporting the value of independent biomarkers, the majority have demonstrated limitations when utilised alone. It is likely that the immunobiology of NSCLC—or any cancer, for that reason—is too nuanced to be effectively evaluated at a univariate level. Further exploration of composite biomarkers, through multi-omic and multi-parametric predictive models that leverage complex computational algorithms to integrate diverse data types, is likely to hold superior predictive power compared to any individual biomarker.

Current strategies to overcome immunotherapy resistance include combination therapy; with dual ICI agents, ICI in combination with chemotherapy, and ICI in combination with radiation. To date, the utilisation of anti-PD-1/PD-L1 ICI alongside novel ICI agents (e.g., targeting LAG-3, TIM-3, TIGIT, and VISTA)—whilst demonstrating synergistic antitumour responses in preclinical studies and early phase clinical trials—have yet to demonstrate improved outcomes in randomised trials of non-selected NSCLC populations. We propose the incorporation of predictive biomarkers into adaptive clinical trial design, where the presence of particular immune signatures is used to guide therapeutics selection.

## Conclusion

In the era of precision oncology, the development and clinical integration of predictive biomarkers in the management of NSCLC is paramount. Continued efforts to unravel immunotherapy resistance mechanisms, refine immune profiling technologies, and incorporate dynamic tumour monitoring into clinical practice will pave the way for a more personalised and efficacious NSCLC treatment paradigm. By tailoring therapies to the molecular profiles of individual patients, we can maximise therapeutic benefit, minimise risk, and ultimately improve NSCLC outcomes on a global scale.
